# *Sarcoptes scabiei*: The Mange Mite with Mighty Effects on the Common Wombat (*Vombatus ursinus*)

**DOI:** 10.1371/journal.pone.0149749

**Published:** 2016-03-04

**Authors:** Kellie Simpson, Christopher N. Johnson, Scott Carver

**Affiliations:** 1 School of Biological Sciences, University of Tasmania, Sandy Bay, Australia; 2 Department of Parks, Primary Industries, Water and the Environment, Hobart, Australia; Université de Sherbrooke, CANADA

## Abstract

Parasitism has both direct and indirect effects on hosts. Indirect effects (such as behavioural changes) may be common, although are often poorly described. This study examined sarcoptic mange (caused by the mite *Sarcoptes scabiei*) in the common wombat (*Vombatus ursinus*), a species that shows severe symptoms of infection and often causes mortality. Wombats showed alterations to above ground behaviours associated with mange. Infected wombats were shown to be active outside of the burrow for longer than healthy individuals. Additionally, they spent more time scratching and drinking, and less time walking as a proportion of time spent above ground when compared with healthy individuals. They did not spend a higher proportion of time feeding, but did have a slower feeding rate and were in poorer body condition. Thermal images showed that wombats with mange lost considerably more heat to the environment due to a diminished insulation layer. Infection status did not have an effect on burrow emergence time, although this was strongly dependent on maximum daily temperature. This study, through the most detailed behavioural observations of wombats to date, contributes to a broader understanding of how mange affects wombat health and abundance, and also to our understanding of the evolution of host responses to this parasite. Despite being globally dispersed and impacting over 100 species with diverse intrinsic host traits, the effects of mange on hosts are relatively poorly understood, and it is possible that similar effects of this disease are conserved in other host species. The indirect effects that we observed may extend to other pathogen types.

## Introduction

The effects of parasites on hosts can be either direct or indirect [1]. Direct effects are physiological changes directly related to infection, such as mounting an immune response, which require energy investment and compete with investment in other physiological processes, leading to life history trade-offs [2, 3, 4]. Indirect effects are impacts that are not an immediate consequence of parasite infection, such as behavioural changes [5, 6, 7, 8]. There is considerable controversy over whether these indirect effects are; (1) of benefit to the host by alleviating some of the negative fitness effects, (2) a ‘side effects’ of pathology with no adaptive value for host or parasite, or (3) whether they are ‘coincidentally beneficial’ to the parasite [9, 10, 11]. Studies of the indirect effects of parasite infection are rare compared to studies of direct effects.

Sarcoptic mange is a globally distributed infectious disease that affects over 100 species in 10 different mammalian orders, including humans [12, 13]. It is caused by the endoparasitic mite *Sarcoptes scabiei*, which exhibits morphological and physiological variation for host specificity [14, 15]. Infection produces two characteristic signs of mange: a thick scaly crust on the epidermis (parakeratosis) that is part of the immune response by the host, and hair loss (alopecia) resulting in large bald patches [14, 16]. These symptoms can take up to three months to develop [12]. Epidermal changes can lead to excoriation caused by scratching and skin fissures, leaving the host susceptible to secondary infections [17]. Direct effects of mange have been well documented in foxes [18], coyotes [19], racoon dogs [20], wombats [16, 17, 21, 22], bobcats [23] and wolves [24, 25].

Although a number of Australian species have been reported to be hosts (for example, [26, 27, 28, 29]), mange is an enzootic disease in wombats [12], affecting two of the three species: the common or bare-nosed (*Vombatus ursinus*) and southern hairy-nosed (*Lasiorhinus latifrons***)** species. Mange is present throughout the common wombat’s entire geographic range [30], and is the most commonly ocbserved disease in both of these species [16]. In high density populations the disease can spread rapidly, resulting in epizootics and local extirpations because of reduced reproduction and increased mortality [12, 13, 30]. The mechanism of transmission of *S*. *scabiei* is not well understood, but is thought to result from sharing of burrows [31], as wombats are characteristically solitary above ground [12, 32]. The mite can survive independently for short periods in suitable environmental conditions, which are thought to occur inside burrows [33].

Some indirect effects of mange on wombats have already been shown, such as reduced perception of the presence of other organisms [32], increased diurnal activity [22, 33] and increased movement distances [32]. These observations suggest that wombats with mange may have higher energy requirements and thus spend more time grazing [12, 17]. On the other hand, it is possible that wombats affected by mange eat less and that they are seen during the day due to blindness caused by scaly skin around the eyes [17]. Another possible explanation could be related to altered thermal tolerance ranges associated with hair loss. Behavioural alterations have been documented in wolves infected by mange, and are possibly related to the loss of thermal energy [25].

This study aimed to investigate indirect effects of *S*. *scabiei* infection on wombats, but also to quantify two known effects from studies of captive wombats, but unstudied in wild populations. We predict that mange infection will have indirect (behavioural) effects on wombats by, (a) causing earlier burrow emergence times relative to healthy wombats, (b) altering above ground behaviour to maximise energy intake and minimise expenditure, and (c) increasing irritation, as determined by scratching (not quantified in wild populations). We also hypothesise that mange severity will cause direct (physiological) effects on their hosts through (a) poorer body condition (also not quantified in wild populations) and (b) increased heat loss to the environment due to hair loss.

## Methods

### Study site

**Fig 1:**
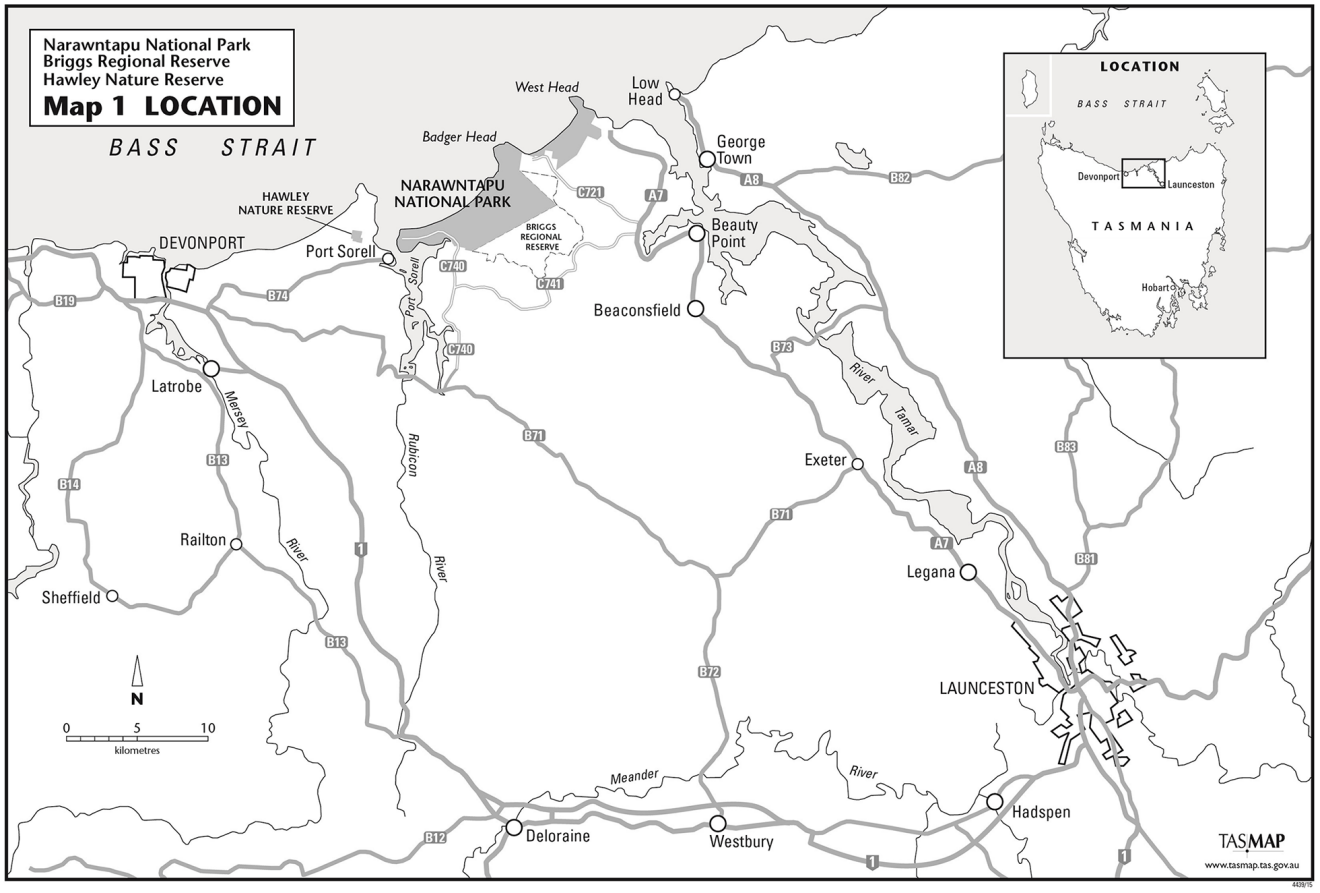
A map of the study site chosen to investigate common wombats (*Vombatus ursinus*) infected with sarcoptic mange. Narawntapu National Park is shaded in grey [42]. Reprinted from [42] under a CC BY license, with permission from the Tasmanian Parks and Wildlife Service, original copyright 2010.

This study took place between March and August of 2014 at Narawntapu National Park on the central north coast of Tasmania (41°07′58″S 146°39′24″E) (Fig 1). Narawntapu is an area of 4,394 hectares, a proportion of which was cleared of vegetation and managed as farmland from 1833 until 1973 when the area was declared a national park [34]. Drainage channels had been dug to divert water from low-lying fields prone to inundation. These fields now support populations of common wombats, as well as Bennett’s wallabies (*Macropus rufugriseus*), grey (Forester) kangaroos (*Macropus giganteus*) and Tasmanian pademelons (*Thylogale billardierii*). The habitat surrounding these large fields includes coastal, wetlands, heathland and remnant dry sclerophyll. The climate is cool temperate maritime climate, with mean rainfall around 750 mm per year, mean January (summer) temperatures of 17°C and mean July (winter) temperatures 9°C [34]. This study was conducted with the permission of the Parks and Wildlife Service, and although permits were sought from the Department of Primary Industries, Parks, Water and the Environment (DPIPWE), they were not required due to the observational nature of the study. All aspects of the study were approved under the University of Tasmania's Animal Ethics Committee under approval number A13809.

Two large paddocks to the left of the Visitors Centre were chosen to be the focus of this study due to easy accessibility, absence of vegetation in wombat grazing areas, a large number of individuals observed grazing and signs of burrow use. Over the last few years mange has swept across the park from east to west and caused population decline (Martin et al. in prep.). In the area chosen for this study, the east section contained wombats affected by mange and the west contained healthy individuals. Consistent with other observations in agricultural landscapes, burrows in this area were located in riparian vegetation [35]. In addition, burrows were also seen in other areas: in drainage channels, throughout the fields and in the elevated sand dunes surrounding the fields containing remnant vegetation. Many of these burrows were no longer in use, likely owing to the decline of the wombat population.

### Mange identification

Wombats were placed in classes of severity of mange based on clinical signs as used in other studies [12, 13, 16, 17, 21, 33]. Each individual was given a mange severity score (diagram modified from [33]—see S1 Text). Each body segment was examined for signs of erythema (skin reddening), parakeratosis (skin thickening) and alopecia (hair loss) and given a score from 0–10, where a score of 0 reflected a healthy segment with no signs of mange and 10 referred to a segment that was ≥70% affected by signs of mange. The overall mange score for each individual was the average of all 14 segments, and is hereafter referred to as mange severity. Individuals were also given a body condition score of ‘very poor’, ‘poor’, ‘good’ and ‘very good’, consistent with body condition scores used in other studies [22, 17]. These definitions reflect the level of protrusion of the ribs, pelvic bones, and shoulder girdle [22, 17]. Animals in very poor condition had all of these elements showing, and animals in very good condition showed very little bone protrusion with large fat reserves. This classification also depended on the appearance of the wombat’s fur [22]. Wombats in very good condition had glossy fur, compared with the dull appearance of animals in poor and very poor condition. Since this study took place, skin scrapings were attained from wombats trapped in the park and these have confirmed the presence of *Sarcoptes scabiei*, and revealed the presence of lice and ticks, although neither of the latter cause hair loss (Carver, pers. obs.).

### Behavioural observations

A total of 20 individual wombats were included in this study, with a single individual observed per day, giving a total of 20 observation days. Identification of individuals was based on a combination of colouring, scars, size, spatial location (territory), mange patterns and burrow fidelity. Animals were observed from an average distance of 10 meters. The exception to this was when attaining calculations of bites per minute and thermal images (both discussed later), as this required short intervals at closer observation distances. If the animal showed signs of disturbance due to human presence, such as pauses during eating, standing still with a raised head, or running (vigilance behaviours), the observation distance was increased. Binoculars were used for observation during daylight hours and at night time we used a Testo (875-2i) high resolution thermal imaging camera with a 2 x telephoto lens. Behaviour was recorded every 30 seconds for as much of the wombat's above ground activity period as possible.

At the time of this study mange-affected individuals were outnumbered by healthy individuals at Narawntapu National Park, thus additional time (1–2 days per individual) on top of the 20 observation days was needed to locate such individuals. Prior to observing a mange affected wombats we walked a circular path around our chosen observation area at one hour intervals, beginning as soon as wombats began emerging to feed (which depended on the time of year, see results). This was done for a minimum of five times in a day. The location, time and identifying features of wombats with mange observed during these walks were recorded on a map to give an indication of an individual’s home range and which burrow systems they were likely to emerge from [31, 36]. For healthy wombats we took an opportunistic approach and waited in the vicinity of areas with a large number of burrows with clear signs of activity [36] and observed the first individual to emerge. This meant that behavioural observations for both healthy and mange-affected wombats usually began as wombats emerged from burrows. Since we needed to observe wombats from a distance, often vegetation (primarily *Lomandra longifolia*) interfered with visibility of entrances of known burrow systems adjacent to grassy fields, and therefore we assumed that observations began within the first 30 minutes of the wombat's emergence.

We attempted to observe wombats for an entire above-ground activity period. If a wombat re-entered a burrow during the observation period, we would wait half an hour for it to re-emerge. Failing this, three half-hourly checks of the area were conducted (with a spotlight at night) to check that the wombat had not re-emerged. If the wombat did re-emerge, behavioural observations would recommence. If the animal did not re-emerge and had previously been above ground for more than six hours, camera traps were set at the burrow entrance or in the general vicinity to confirm that it did not re-emerge before dawn.

During observation, we recorded the particular behaviour of the wombat for the majority of each 30-second interval, classified as ‘walking’, ‘drinking’, ‘feeding’, ‘running', ‘standing still’, ‘sitting’, ‘sleeping’, ‘defending territory’ and ‘digging’. The first three behaviours accounted for more than 95% of behaviours. A distinction was made between 'feeding' and 'walking'. Wombats often take steps forward when feeding; therefore we considered a wombat to be 'walking' when it had taken more than four consecutive steps. Each 30 second time interval in which an individual scratched was also recorded (and converted into ‘percentage of 30 second intervals scratching’), as this behaviour rarely accounted for the majority of the 30 second observation period. Wombats mostly scratched using their feet, but we also included rubbing against objects such as tree trunks and fence posts [36]. Additionally, feeding rate was recorded by calculating the number of bites taken in one minute.

### Thermal images

A Testo (875-2i) high resolution thermal imaging camera was used to take images of wombats under observation. This particular camera has an accuracy range of ± 2% of the temperature reading. One set of images was taken soon after burrow emergence (once the individual had settled to graze) and another was taken several hours later. For individuals that were active during the day and moved into a burrow around sunset, both sets of images were under day-time conditions, but for others one set of images was taken in the day time and one set after dark. Images were attained for most individuals (n = 14), but unfortunately a small number (n = 7) were particularly sensitive to human presence and showed signs of disturbance when we attempted to get closer. Attempts to obtain images in these cases were abandoned due to risk of losing the individual after disturbance. Care was taken to avoid the effects of solar radiation, by photographing the individual from its shady side, or by attaining images when cloud blocked direct solar radiation.

### Statistical analyses

All analyses were conducted in R (www.r-project.com), version 3.1.1. Healthy wombats were those that had a mange severity score of 0 and wombats with mange were determined to be those with mange severity scores greater than 0.

#### a) Indirect effects (behavioural)

To examine the effects of mange on emergence time, a multiple regression was used with mange status and daily maximum temperature as predictors, and wombat emergence time as the response variable. This model also included an interaction between mange and maximum daily temperature. Daily maximum temperature measurements were obtained from Devonport Airport (Australian Bureau of Meteorology), which is the closest weather station to Narawntapu National Park. To rule out any confounding weather effects, humidity and rainfall measures for the days that observations took place were also evaluated in preliminary analyses. However, owing to these results not showing any effect, we kept the analyses focussed on temperature as the single climatic variable.

#### b) Direct effects (physiological)

Simple linear regressions were used to assess the relationship of mange severity with irritation, and also mange severity with body condition. Since body condition was a dependent variable in this analysis, the condition categories were converted to a scoring system (very poor = 0, poor = 1, good = 2 and very good = 3). To assess whether hair loss leads to increased heat loss to the environment, pictures taken with the thermal imaging camera were examined using the Testo IRSoft software (www.testo.com/irsoft), version 3.3. This camera takes low quality ‘real’ image in addition to a thermal image, and both of these photos were compared side by side to exclude any effects of solar radiation. To measure the temperature differentials between the wombat and the surrounding ambient temperature, five random points were taken from the background of the thermal images to obtain the average ambient temperature. The hottest point on the wombat’s body (excluding the head) was obtained using the ‘hot spot’ function. This gave a temperature differential between the ambient temperature and wombat body temperature. A mixed effects model then used temperature differential and the time of day the photo was taken as fixed effects. The effect of average ambient temperature was controlled for by inclusion as a random effect. A likelihood ratio test was then used to calculate the P value. In preliminary analyses we explored the effect of accounting for time as a circular variable and also removing thermal images taken after midnight to control for this. Overall the conclusion remained the same and thus we elected to stick to a linear effects model for simplicity. We also evaluated if our analyses might be complicated by a correlation between time of day and the average ambient temperature, but found this not to be the case over the temporal span of our sampling (Spearman correlation ρ = 0.182, P = 0.418).

## Results

### Indirect effects (behavioural)

**Fig 2:**
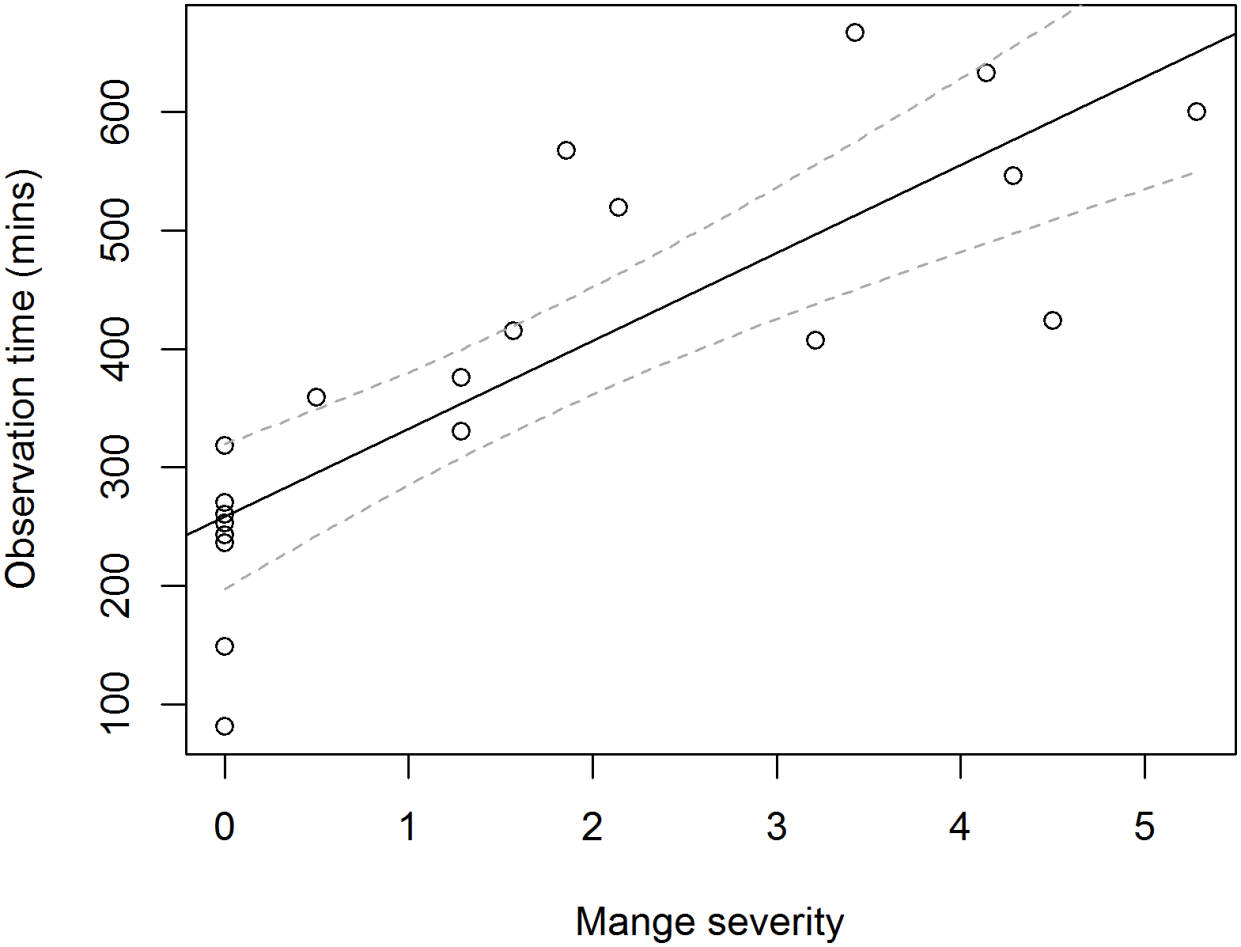
The observation time of common wombats (*Vombatus ursinus*) is associated with the intensity of mange infection (mange score) at Narawntapu National Park in Tasmania (F_(1,18)_ = 38.8, P<0.001).

A total of 125 hours was spent observing 20 different individuals. We considered this sample size to be relatively large for a free-ranging population. Of these, 8 (40%) were judged to be healthy (mange severity score 0) and the other 12 (60%) were in various stages of mange infection. Of those affected by the disease, mange scores ranged from 0.5–5.2 and no individuals with 'very severe' mange were located at the time of this study. There was variation in the time each individual was observed (Fig 2) and a strong relationship between mange severity and observable time above ground. Wombats with mange were observed for between 330–667 minutes, whereas healthy wombats were observed for between 81–318 minutes. However, it should be acknowledged that individuals with mange were easier to observe as they were less easily disturbed [17], and remained in the open for long intervals. Further, mange affected individuals were easily identified, due to their distinctive mange patterns. Healthy wombats on the other hand, had to be observed from a greater distance in order to prevent disturbance and had only subtle differences between individuals that were not easily seen at night. Despite this difficulty in capturing whole activity periods, healthy wombats were observed for an average of 210 minutes (3.5 hours) which is consistent with activity periods in other studies (for example [36]), suggesting that the majority of their above ground behaviours were captured by our observations. To control for this variation in observation times, behavioural observations are expressed as percentages of time that an individual exhibited a particular behaviour during its above ground activity time, rather than total amounts of time.

**Fig 3:**
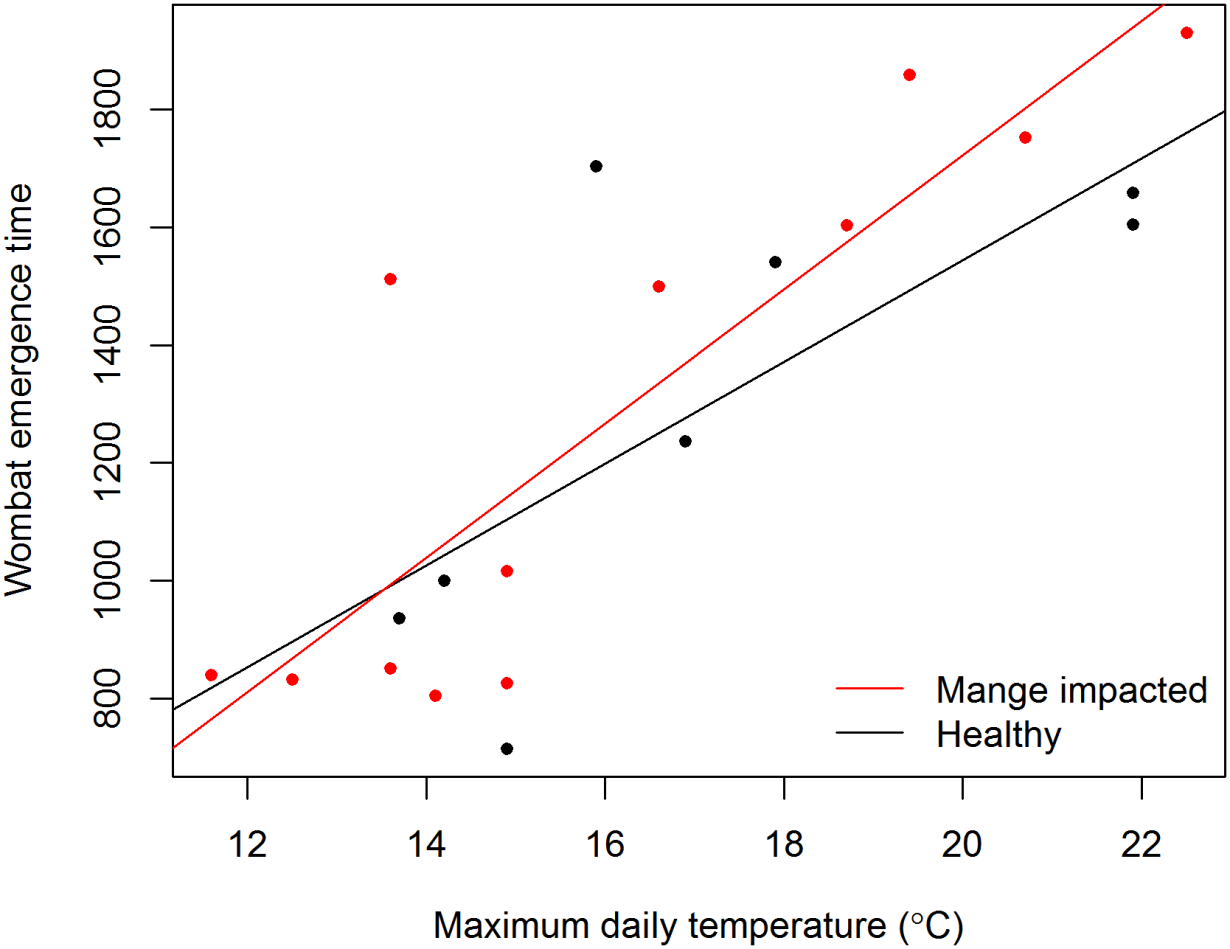
The burrow emergence time of 20 common wombats (*Vombatus ursinus*) recorded from March-August 2014 at Narawntapu National Park Tasmania. Emergence time is not altered by sarcoptic mange (*Sarcoptes scabiei*), but is strongly correlated with daily maximum temperature. Confidence intervals around regression lines were omitted for clarity of image.

The majority of the wombats that were followed in this study were animals that emerged in the first ‘wave’ of individuals to emerge from the burrows to feed. The timing of this wave of emergence was related to maximum daily temperature (Fig 3), being later as temperature increased (Mange status F_1_ = 0.031, P = 0.862; maximum temperature F_1_ = 41.161, P<0.001; mange status*maximum temperature F_1_ = 0.721, P = 0.408, Error df = 16). There was no difference in mean time of emergence of healthy and mange-affected individuals, nor was there a significant interaction between mange and maximum temperature on emergence time.

**Fig 4:**
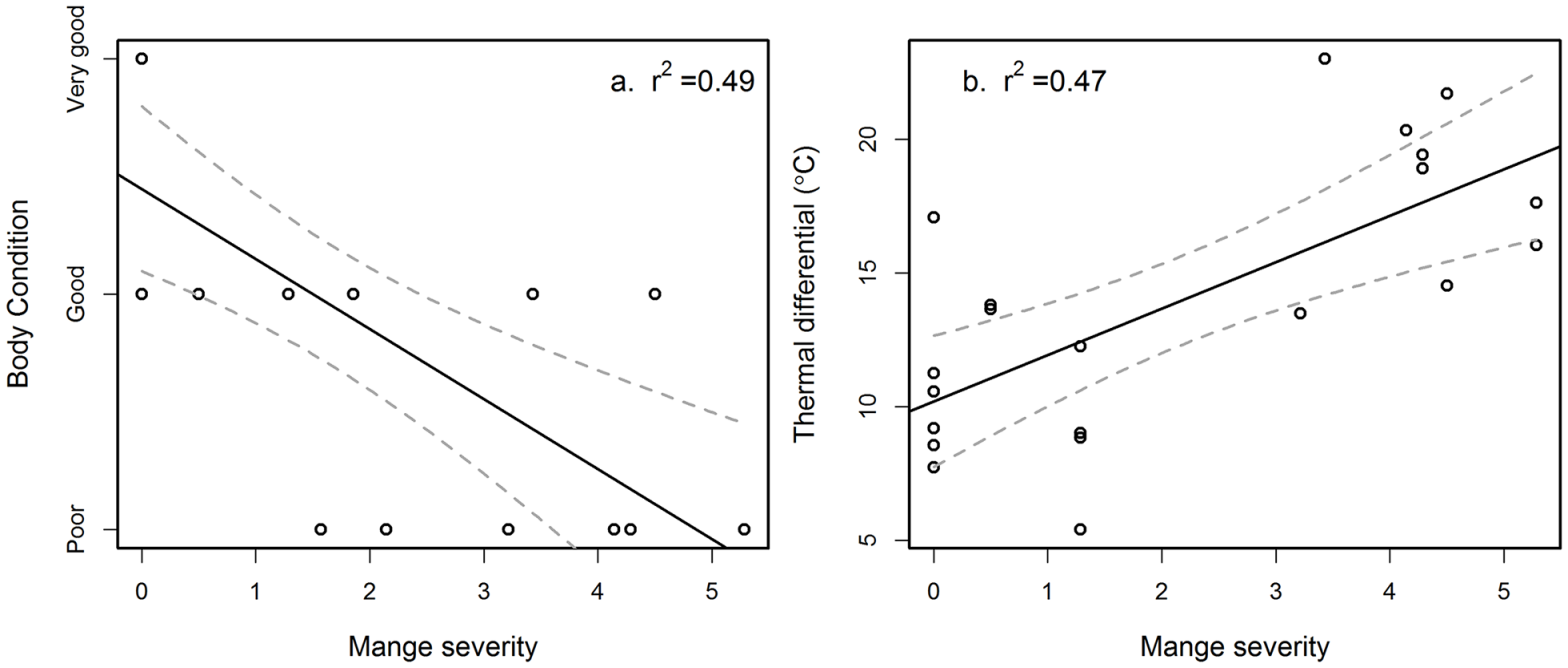
The significant indirect effects of sarcoptic mange on the behaviour of common wombats (*Vombatus ursinus*). Wombats infected by mange exhibit changes to time allocations to above ground behaviours: (a) they spend a higher percentage of time drinking water, (b) a lower percentage of time walking, (c) have a slower feeding rate and (d) higher percentage of 30 second time intervals scratching.

Our observations confirmed that wombats infected with mange exhibit different time allocation to above ground behaviours compared with healthy individuals. Wombats with mange spent a higher percentage of time drinking (F_1,18_ = 6.304, P = 0.022) and scratched more frequently (F_1,18_ = 5.85, P = 0.026), although they spent a smaller percentage of time walking (F_1,18_ = 12.77, P = 0.002) when compared with healthy wombats (Fig 4). They also had a slower feeding rate (F_1,8_ = 6.207, P = 0.031). However, contrary to predictions, wombats with mange did not differ from healthy wombats in the percentage of time feeding (F_1,18_ = 0.031, P = 0.721).

### Direct effects (physiological)

**Fig 5:**
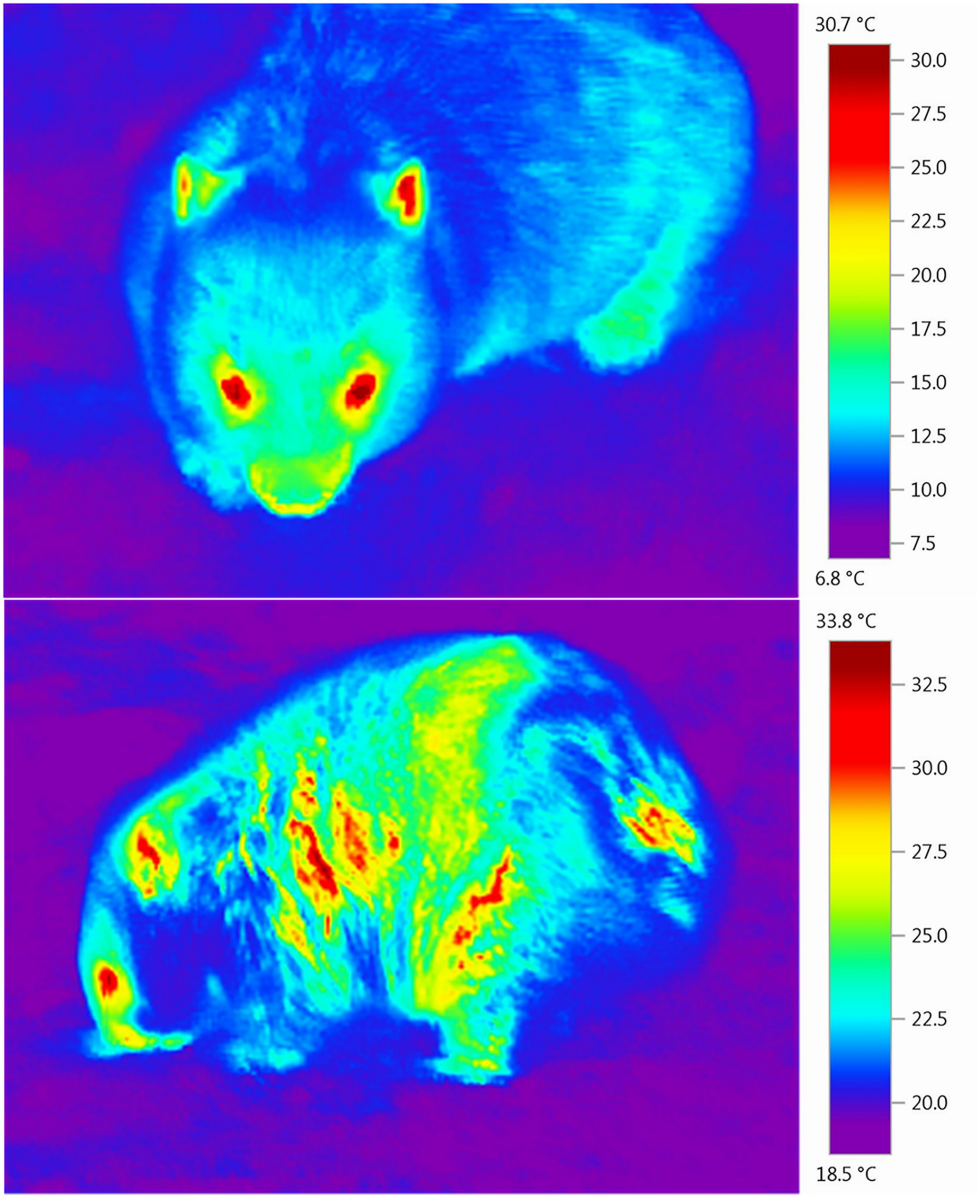
The effect of mange on common wombats (*Vombatus ursinus*) at Narawntapu National Park in Tasmania. On the left (a) loss of body condition (F_(1, 18)_ = 18.4, P<0.001) and on the right (b) loss of heat to the environment as represented by temperature differential (Z = 8.99, P<0.001).

**Fig 6:**
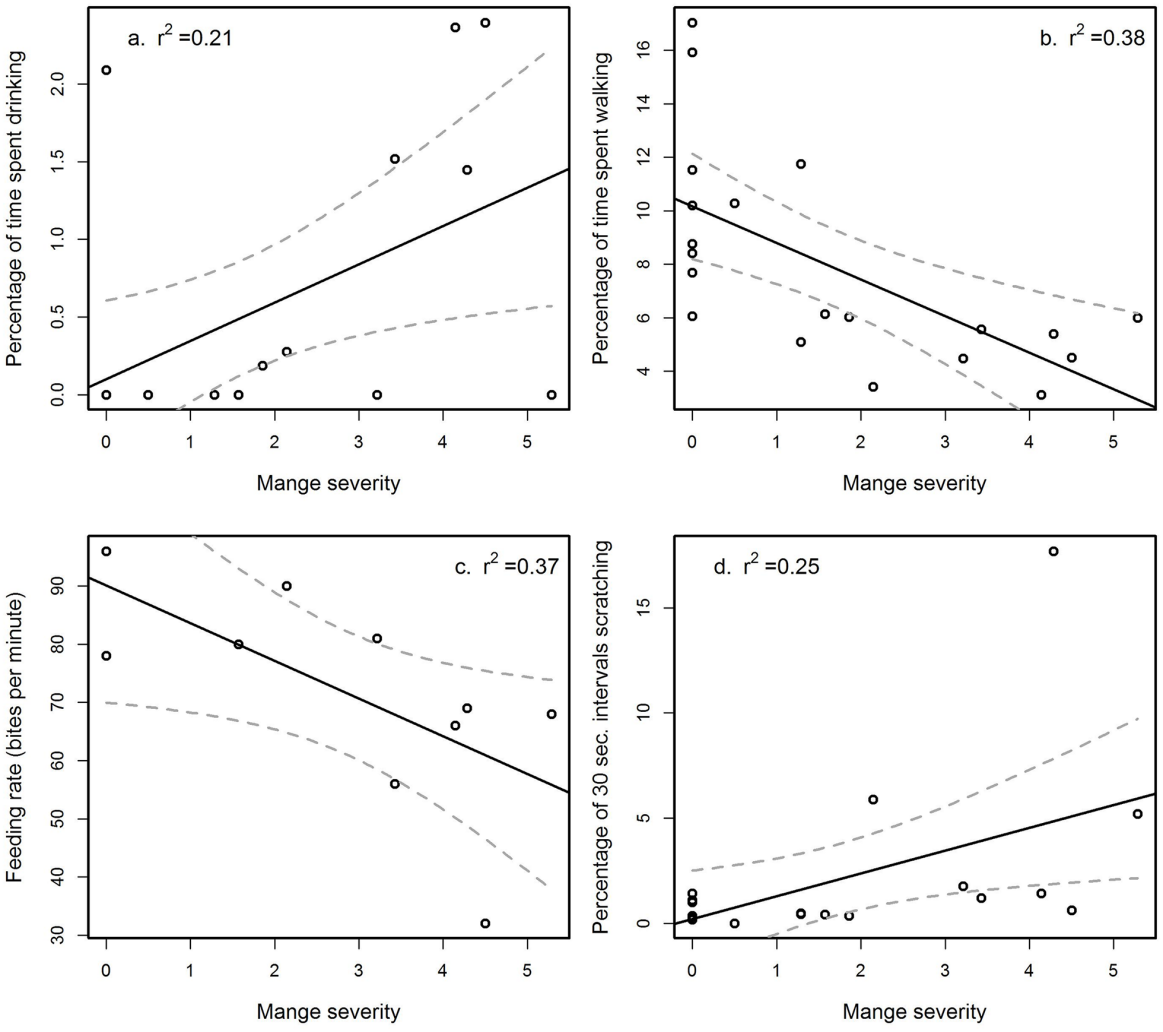
Thermal images of common wombats (*Vombatus ursinus*) taken with a Testo (875-2i) high resolution thermal imaging camera with a 2x telephoto lens. Shows a healthy wombat (top) and a wombat exhibiting signs of sarcoptic mange (bottom), a disease caused by the S*arcoptes scabiei* mite. Note the differences in the thermal profile between the two images.

Wombats with mange had poorer body condition (Fig 5a). Most wombats fell into the categories of 'good' and 'poor' rather than 'very good', and none were noted as 'very poor'. Thermal images of healthy wombats had a relatively uniform thermal profile across their bodies (Fig 6). Thermal images of wombats with mange showed a patchy profile in comparison. In areas with hair loss, the exposed skin was much higher in temperature than the rest of the body. Wombats with mange had a significantly higher temperature differential between the ambient temperature and their body temperature indicating that they are more susceptible to heat loss to the environment (Fig 5b). Temperature differential was unrelated to the time of day at which the image was taken (Z = 0.79, P = 0.433), owing to the relatively narrow ambient temperature range in which thermal images were taken.

## Discussion

The aim of this study was to investigate the effects of parasites on hosts, focussing on sarcoptic mange in common wombats. Contrary to our predictions, burrow emergence time of wombats with mange did not differ from healthy wombats. Although there was no significant effect of mange, there was a strong relationship between emergence time and daily maximum temperature. We also hypothesised that wombats affected by mange would show different time allocations to above ground behaviours compared with healthy wombats, as a result of indirect (behavioural) effects of the parasite. Mange altered movement, feeding rate, drinking and irritation behaviours, but had no effect on the percentage of time spent feeding. Finally, we hypothesised that mange would have direct effects on wombats through a reduction in body condition and also increased heat loss to the environment. Mange affected wombats did indeed have poorer body condition and a greater temperature differential between the ambient temperature and body temperature.

Although we found no difference in the burrow emergence time or in percentage of time spend feeding, we did see a difference in the above ground observation times between healthy and mange affected wombats. Our results indicate that wombats with mange were active outside of the burrow for longer periods than healthy wombats, which could be due to the difficulties we had in observing healthy individuals, although we think it is more likely suggestive of the disease leading to increased total foraging time in mange affected wombats. If our observation times do accurately reflect activity periods, wombats with mange could be increasing total foraging time to offset the increased immunological and thermal costs, but unlike wombats on the mainland, the increased time could coincide with the end of the activity period, rather than the beginning. Further studies could investigate this, particularly during winter when overnight temperatures drop considerably, leaving wombats with the disease vulnerable to greater thermal costs.

### Indirect effects (behavioural)

Previous anecdotal evidence suggested wombats with mange exhibit increased diurnal activity [12, 17, 33]. However, our study found no such relationship, but did indicate an effect of mange severity on total above-ground observation time. Increased diurnal activity is proposed to be due to the need to meet increased nutritional demands by emerging earlier to allow for longer foraging periods [12, 33]. Alternatively, diurnal activity could be explained by blindness and loss of light recognition caused by epidermal thickening around the eyes [12, 17]. The differnce between our study and others may owe to regional differences in wombat behaviour or methodological approaches of studies (namely observation of entire above-ground periods). A previous study monitoring activity using camera traps found wombats detected during the day were more likely to have mange, and also that wombats with mange were generally detected when the air temperature was higher [33]. Although this study was able to obtain over 900 images, it was limited in its capacity to identify individuals. In addition to this, it captured only a snap shot of a wombat's activity period and was not able to accurately determine burrow emergence times. We suggest that our study methodology may address the effect of mange on emergence behaviour more precisely, and that regional comparisons of emergence behaviour owing to this disease would be valuable.

Interestingly, wombats in this study, and indeed across Tasmania, are not strictly nocturnal [36]. Wombat emergence on the Australian mainland is restricted by warm temperatures [37]. In Victoria wombats were shown to emerge at consistent times at or just after dusk in winter and in summer, even though there are large differences in ambient temperatures between the seasons [38]. Additionally, wombats in NSW have peaks of activity at the beginning and end of each night [31]. It appears that wombats in Tasmania may not be as restricted in their emergence times. In Tasmania they are often seen out in the day, particularly in the north-east of the state, where they have been reported to emerge as early as 10 am in cooler months [36]. This study found the highest increase in diurnal activity during the colder winter months, when maximum daily temperature was low, with wombats emerging as early as 7:15 am. The literature often refers to wombats as being 'generally nocturnal' rather than being strictly 'nocturnal' animals, and this study suggests 'generally nocturnal' may be synonymous with 'temperature dependent activity'.

Wombats in this study spent a higher proportion of time drinking as mange severity increased. There is some evidence that mange can cause dehydration. Abnormalities in the blood that suggest reduced kidney function of other host species have been reported, such as racoon dogs [20, 39], coyotes [19] and wombats [17]. Raccoon dogs (*Nyctereutes procyonoides*) infested with *S*. *scabiei* had significant increases in blood urea nitrogen (BUN), lower calcium and higher phosphorous [39], possibly due to starvation or reduced perfusion in the kidneys. There was also an increase in BUN in coyotes (*Canis latrans*) suffering from mange [19]. In humans, reduced perfusion is associated with increased thirst. Increased BUN could be the cause of increased drinking by wombats with mange, but this could also be a consequence of water loss through open wounds. Increased dehydration occurs in humans with open wounds [40]. These two explanations for increased drinking are not mutually exclusive and further investigation is needed to link underlying physiological processes with this change in behaviour.

Wombats with more severe mange spent a lower proportion of time walking. This decrease suggests that wombats with mange may be reducing their energy expenditure associated with movement as a trade off against fighting infection. Two gray wolves (*Canis lupus*) with mange have been documented as having more concentrated movements and comparatively higher proportions of road travel, suggesting that this was related to their poor physical condition [25]. It is also possible that wombats with mange walk less due to the pain caused by fissuring of the skin in moderate and severe cases of mange [32]. The lower proportion of time spent walking observed in this study was initially thought to be compensated by an increase in proportional feeding behaviours. However, this was not the case and it appears that wombats are instead offsetting this proportion of time through increased scratching and drinking.

Empirical studies have suggested that wombats with mange may have increased nutritional demands associated with mounting an immune response [12, 17]. This study found no effect of mange severity on the percentage of time spent feeding, but mange affected wombats did show a slower feeding rate (bites per minute), which supports evidence that wombats with mange may have impaired powers of mastication [17]. This could reflect the higher energy constraints of wombats with mange. This study could not conclusively show that mange has an impact on total foraging time, owing to the difficulties with capturing entire activity periods (specifically the time that they returned and stayed within burrows). However, our observation times of healthy and mange affected wombats are strongly suggestive of this (see [33] and [36] for typical activity periods of healthy wombats). This study does show that wombats with mange do not emerge earlier than healthy wombats, therefore in Tasmania, if they are spending longer periods of time foraging, this would be added on to the end of their activity period rather than the beginning.

We found that wombats with mange spent more time scratching. The relationship between scratching and mange has been quantified once on a small sample (n = 9) of captive wombats, showing that experimentally infected individuals spent more time scratching than healthy controls (as a percentage of 10 minutes) [16], and that the amount of time that an animal spent scratching was not correlated with thickness of the epidermis or the intensity of infection [16]. We found relatively large inter-individual variability in the percentage of time spent scratching among the mange-affected wombats, but little variation was observed among healthy wombats.

More broadly, anti-parasite grooming activity, such as scratching, is an energetic cost to the host [41]. This is a response to cutaneous irritation may have some benefits for parasite removal in the early stages of infection of larger parasites, but in the case of mange, scratching is likely to be ineffective against the microscopic *Sarcoptes scabiei*, therefore scratching is likely attributed to irritation. Grooming behaviour has been linked to a significant increase in metabolic costs in mouse-eared bats (*Myotis myotis*) [41]. They also found that the bats lost body condition, and as they increased the proportion of time spent grooming the proportion of time spent resting decreased. In wombats, the energetic cost of scratching is likely to lead to further costs, as scratching causes hair loss (in addition to that already caused by the parasite) and excoriation (loss of skin) [16], which leads to reduced capacity of the wombat's insulation layer and likely effects their thermoregulatory ability (discussed below), as well as increased susceptibility to secondary infections in skin fissures [17].

### Direct effects (physiological)

Wombats with more severe mange were in poorer body condition than healthy individuals, as also shown in other studies [22, 16, 17], suggesting that the disease may have caused them to utilise their energy reserves in order to meet energy requirements that exceed their nutritional supply [17]. Loss of body condition has also been reported in foxes [18] and coyotes [19] with sarcoptic mange, although there was only a small change in body condition in Iberian wolves [24]. Reduction in body condition is also seen in other host-parasite systems and can affect host vulnerability to secondary infections [42].

Further, the body condition of a host affects its ability to mount an immune response (known as its immunocompetence). This has been discussed in the literature as ‘condition dependence’ [43, 44, 45]. An individual in good condition has more resources and therefore a greater capacity to mount an immune response, which improves its chances of survival [40]. Healthy wombats at Narawntapu appeared to be in good body condition, which means that at initial infection they may have a greater capacity for fighting the disease, although as the disease progresses, loss of body condition may coincide with reduced immunocompetence.

Wombats occupy a unique ecological position due to a combination of traits that may also limit their immunocompetence against mange. As large, burrowing herbivores, wombats have a restrictive energetic ecology [46]. The energetic cost of burrowing is accentuated in larger animals compared with smaller mammals, as it cannot be immediately compensated [46]. Wombats are the only extant mammals above 10 kg to regularly construct burrows [46]. In addition to this, wombats are restricted by their low energy herbivore diet, although they possess a number of morphological adaptations that allow them to conserve energy [46, 47]. They have a low metabolic rate for their size [47, 48] and are highly adapted for digging [46]. They also have modifications to the digestive system that allow them to utilise poor quality food (predominately grass [49]), such as colonic fermentation, continuously growing teeth and a large gut capacity relative to body weight that results in long food retention time [46, 50, 51]. These adaptations allow wombats to have low energy expenditure and smaller home ranges relative to their body weight [31, 46], occupy poor quality habitat and spend less time feeding [48]. This restricted energetic ecology combined with parasite-driven changes to energy expenditure may explain why mange has a pronounced effect in wombats. If this is the case, wombats infected with mange should show noticeable indirect effects of parasite infection that reflect energy constraints. Their responses to infection should indicate changes in energy allocation, as a result of strategies to reduce the negative fitness effects of parasite infection, but also show energy conservation strategies.

This is the first study to definitively show that wombats with increased severity of mange are subject to greater heat loss to the environment. This has not been shown in any other host species affected by mange, although rock doves (*Columbia livia*) infected with feather-feeding lice were found to have increased thermal conductance [52]. The thermal images indicated that wombats with mange have diminished insulation owing to fur loss across the body. In addition to this, *S*. *scabiei* causes inflammation of the skin, causing it to become swollen, red and hot [17] and results in greater blood perfusion to infected sites which could speed up the heat loss process. Wombats with mange, therefore, are likely to have greater energetic costs associated with thermoregulation and this may be linked with changes in behaviour observed in this study, although this would require further examination.

In the case of the two gray wolves previously mentioned, the observed behavioural changes (concentrated movements, long periods of inactivity and concentrated travel on roads) could be attributed to impaired thermoregulatory ability [25], since additional energy needs to be expended on thermoregulation in infected individuals and these costs compound over time and contribute to poor body condition [46]. Their behaviour could be regarded as energetically conservative. Future studies could investigate direct links between heat loss and behavioural changes that have been suggested in the literature [25], to disentangle whether these behaviours are a functional response to thermoregulation demands or a consequence of pathogenesis. Manipulation experiments whereby healthy hosts have their hair trimmed to reduce their insulation layer and behaviours followed could address these links.

Owing to increased heat loss, wombats with mange could have a different optimal temperature range compared with healthy wombats. Wombats on the Australian mainland are more restricted by higher temperatures than wombats in Tasmania, and loss of large patches of fur may alleviate some of these temperature constraints. This would allow them to emerge earlier and could explain why wombats with mange have been observed above ground during the day [33]. This could also explain why wombats are seen less after midnight when temperatures are colder [33], as the cost of thermoregulation at lower temperatures is greater. This underlying mechanism has not been investigated and it is possible that there are multiple non-mutually exclusive processes occurring. While the results of this study suggest energetic costs associated with the direct and indirect effects of mange, metabolic rates were not directly measured.

## Conclusions

The aim of this study was to investigate and clarify known and unknown effects of sarcoptic mange on wombats. The main component of this study consisted of comprehensive behavioural observations of a wild population. Thermal images were also taken to determine whether wombats with mange are subject to greater heat loss to the environment. It was found that there was no effect of mange on emergence time, although this was strongly related to daily maximum temperature. Several behavioural alterations were attributed to mange severity, such as an increased proportion of time spent drinking, a lower proportion of time spent walking and slower feeding rate. On the other hand, there was no effect of mange on the proportion of time spent feeding, despite predictions in the literature. Wombats with more severe mange spent more time scratching due to irritation of *S*. *scabiei*, had poorer body condition and were subject to greater heat loss to the environment due to hair loss caused by the disease. Results indicate that mange affected wombats were also active for longer periods outside of the burrow than their healthy counterparts. This study provides the most comprehensive behavioural observations of wombats from a wild population and was able to demonstrate effects of mange on a host with significant energy constraints that have not been demonstrated elsewhere. This study provides a basis for future studies that could investigate whether the same effects occur in other host species that are impacted by mange, but also to investigate whether these effects apply to other host-parasite interactions.

## Supporting Information

S1 Table Dataset underlying study findings of the direct and indirect effects of sarcoptic mange on common wombats (*Vombatus ursinus*). (DOCX)

S1 Text Diagram used to record mange serverity in common wombats (*Vombatus ursinus*) at Narawntapu National Park, Tasmania. Each segment was allocated a number (0–10) and the average of all segments gave the overall mange serverity score for each individual. Based on diagram in [33].
(TIF)
