# Correction: *Sarcoptes scabiei*: The Mange Mite with Mighty Effects on the Common Wombat (*Vombatus ursinus*)

**DOI:** 10.1371/journal.pone.0153997

**Published:** 2016-04-14

**Authors:** The PLOS ONE Staff

The images for Figs 4, 5 and 6 appear in the incorrect order in the published article. The image that appears as Fig 4 should be Fig 5, the image that appears as Fig 5 should be Fig 6, and the image that appears as Fig 6 should be Fig 4. The captions appear in the correct order. Please see the correct Figures and their captions here. The publisher apologizes for the errors.

**Fig 4:**
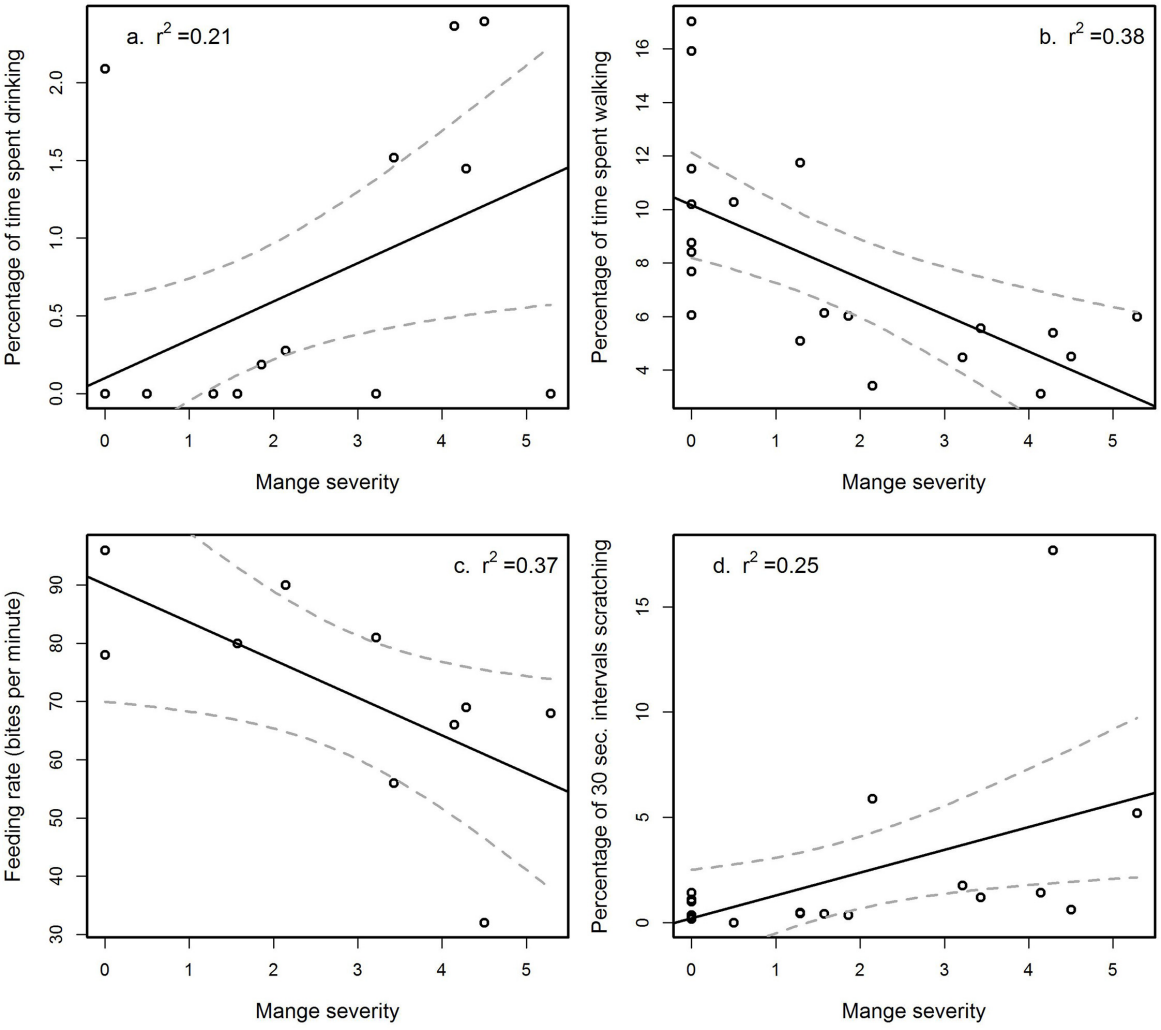
The significant indirect effects of sarcoptic mange on the behaviour of common wombats (*Vombatus ursinus*). Wombats infected by mange exhibit changes to time allocations to above ground behaviours: (a) they spend a higher percentage of time drinking water, (b) a lower percentage of time walking, (c) have a slower feeding rate and (d) higher percentage of 30 second time intervals scratching.

**Fig 5:**
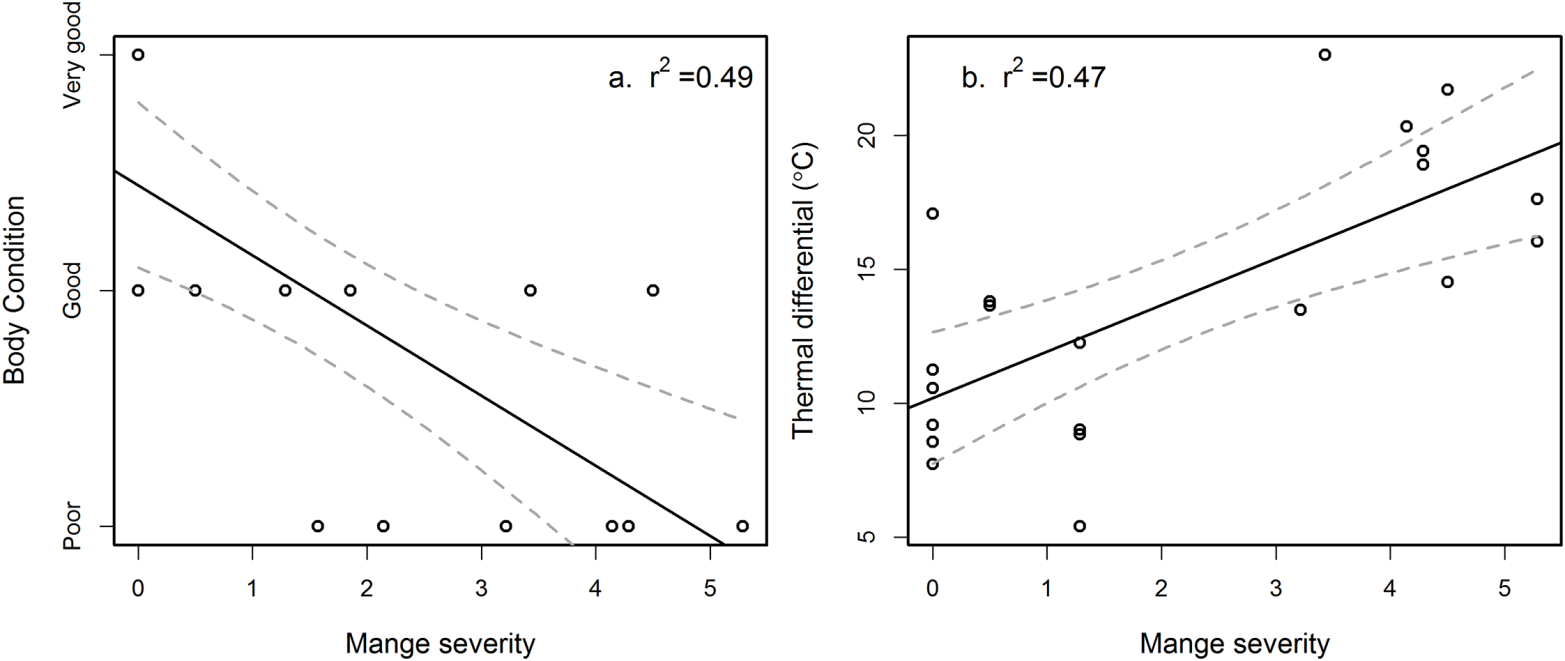
The effect of mange on common wombats (*Vombatus ursinus*) at Narawntapu National Park in Tasmania. On the left (a) loss of body condition (F(1, 18) = 18.4, P<0.001) and on the right (b) loss of heat to the environment as represented by temperature differential (Z = 8.99, P<0.001).

**Fig 6:**
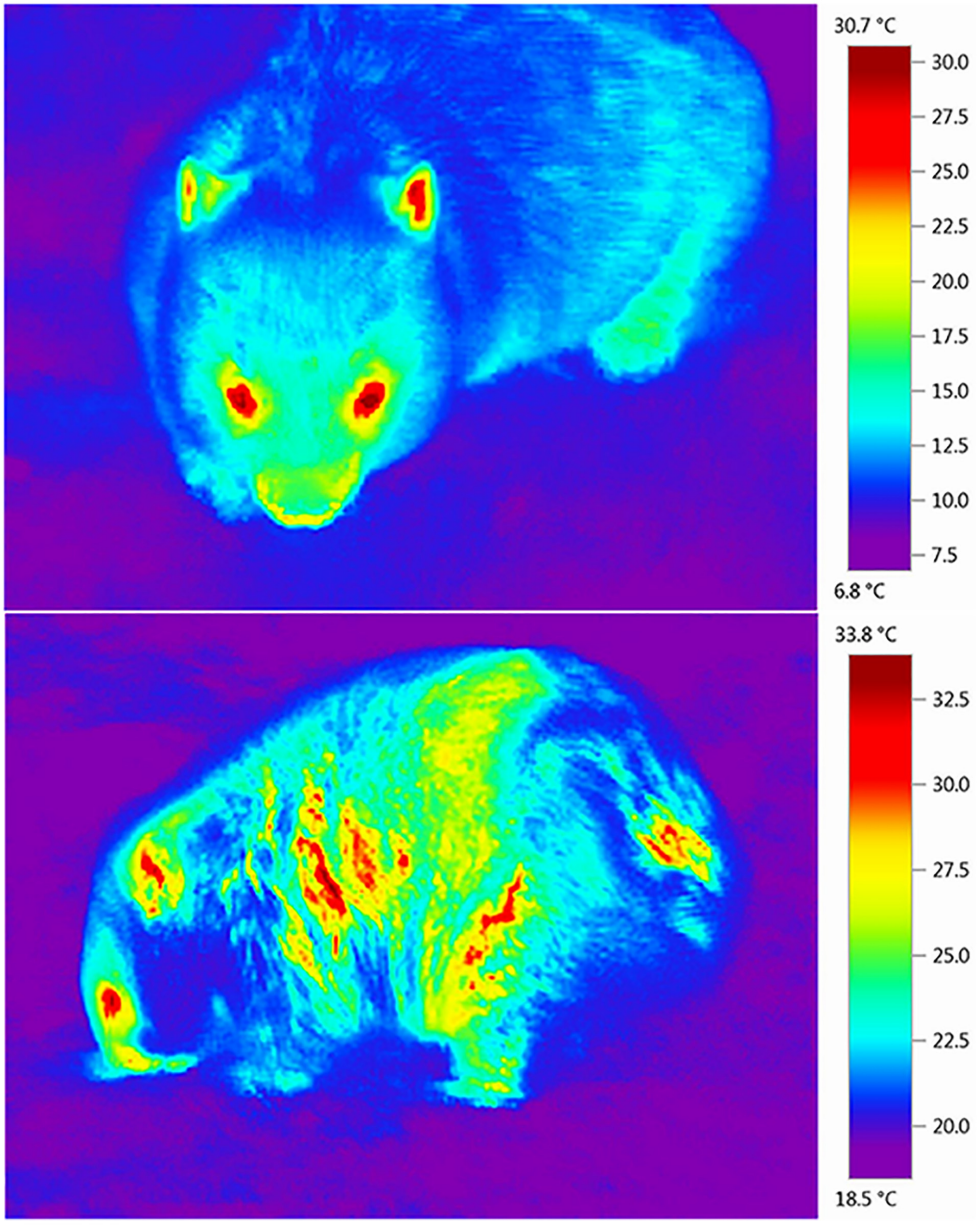
Thermal images of common wombats (*Vombatus ursinus*) taken with a Testo (875-2i) high resolution thermal imaging camera with a 2x telephoto lens. Shows a healthy wombat (top) and a wombat exhibiting signs of sarcoptic mange (bottom), a disease caused by the *Sarcoptes scabiei* mite. Note the differences in the thermal profile between the two images.
