# Supplementary material for: Sarcoptes scabiei: The Mange Mite with Mighty Effects on the Common Wombat (Vombatus ursinus)
Source: PLoS One. 2016 Mar 4;11(3):e0149749. doi: 10.1371/journal.pone.0149749 (PMC4778766; doi:10.1371/journal.pone.0149749)
Supplement: S1 Table — (DOCX) [file pone.0149749.s001.docx]

| ID | Maximum daily temperature (°C) | Mange severity score | Body condition | Emergence time (24 hour) | Observation time (minutes) | Feeding rate (bites/minute) | % time walking | % time drinking | % time feeding | % time 30 second intervals scratching | Time of thermal image (24 hour) | Temperature differential (°C) |
| --- | --- | --- | --- | --- | --- | --- | --- | --- | --- | --- | --- | --- |
| 1 | 22.5 | 4.5 | Good | 1930 | 424 | 32 | 4.5 | 2.4 | 91.8 | 0.6 | 1956 | 14.5 |
| 2 | 20.7 | 1.3 | Good | 1753 | 330 |  | 5.1 | 0.0 | 93.4 | 0.5 | 1851 | 12.3 |
| 3 | 19.4 | 0.5 | Good | 1859 | 359 |  | 10.3 | 0.0 | 88.2 | 0.0 | 1720 | 13.8 |
| 4 | 21.9 | 0.0 | Very Good | 1605 | 149 |  | 15.9 | 0.0 | 84.1 | 1.0 | 1601 | 9.3 |
| 5 | 21.9 | 0.0 | Very Good | 1659 | 318 | 78 | 10.2 | 0.0 | 86.4 | 0.2 | 1901 | 8.6 |
| 6 | 15.9 | 0.0 | Very Good | 1704 | 260 |  | 8.4 | 0.0 | 73.8 | 0.3 | 1807 | 11.3 |
| 7 | 17.9 | 0.0 | Very Good | 1541 | 81 | 96 | 6.1 | 0.0 | 93.3 | 0.4 |  |  |
| 8 | 18.7 | 1.3 | Good | 1604 | 376 |  | 11.8 | 0.0 | 76.1 | 0.5 | 1839 | 5.4 |
| 9 | 16.6 | 5.3 | Poor | 1500 | 600 | 68 | 6.0 | 0.0 | 84.6 | 5.2 | 1645 | 16.0 |
| 10 | 13.6 | 4.3 | Poor | 1512 | 546 | 69 | 5.4 | 1.4 | 89.0 | 17.7 | 1924 | 19.4 |
| 11 | 16.9 | 0.0 | Very Good | 1237 | 270 |  | 17.0 | 0.0 | 80.9 | 1.1 |  |  |
| 12 | 13.6 | 2.1 | Poor | 0851 | 519 | 90 | 3.4 | 0.3 | 91.1 | 5.9 |  |  |
| 13 | 14.2 | 0.0 | Good | 1000 | 253 |  | 7.7 | 0.0 | 91.7 | 1.4 | 1753 | 17.1 |
| 14 | 14.9 | 3.4 | Good | 1016 | 667 | 56 | 5.6 | 1.5 | 92.3 | 1.2 | 1744 | 23.0 |
| 15 | 11.6 | 4.1 | Poor | 0840 | 633 | 66 | 3.1 | 2.4 | 78.4 | 1.4 | 1549 | 20.3 |
| 16 | 12.5 | 3.2 | Poor | 0832 | 407 | 81 | 4.5 | 0.0 | 95.0 | 1.8 | 0930 | 13.5 |
| 17 | 14.1 | 1.6 | Poor | 0805 | 415 | 80 | 6.1 | 0.0 | 94.0 | 0.4 |  |  |
| 18 | 14.9 | 0.0 | Good | 0715 | 236 |  | 11.5 | 0.0 | 93.7 | 0.3 |  |  |
| 19 | 14.9 | 1.9 | Good | 0826 | 567 |  | 6.0 | 0.2 | 93.6 | 0.4 |  |  |
| 20 | 13.7 | 0.0 | Good | 0936 | 243 |  | 8.8 | 2.1 | 88.9 | 0.4 | 1016 | 9.2 |
