# Supplementary figures and images for: Sarcoptes scabiei: The Mange Mite with Mighty Effects on the Common Wombat (Vombatus ursinus)
Source: PLoS One. 2016 Mar 4;11(3):e0149749. doi: 10.1371/journal.pone.0149749 (PMC4778766; doi:10.1371/journal.pone.0149749)

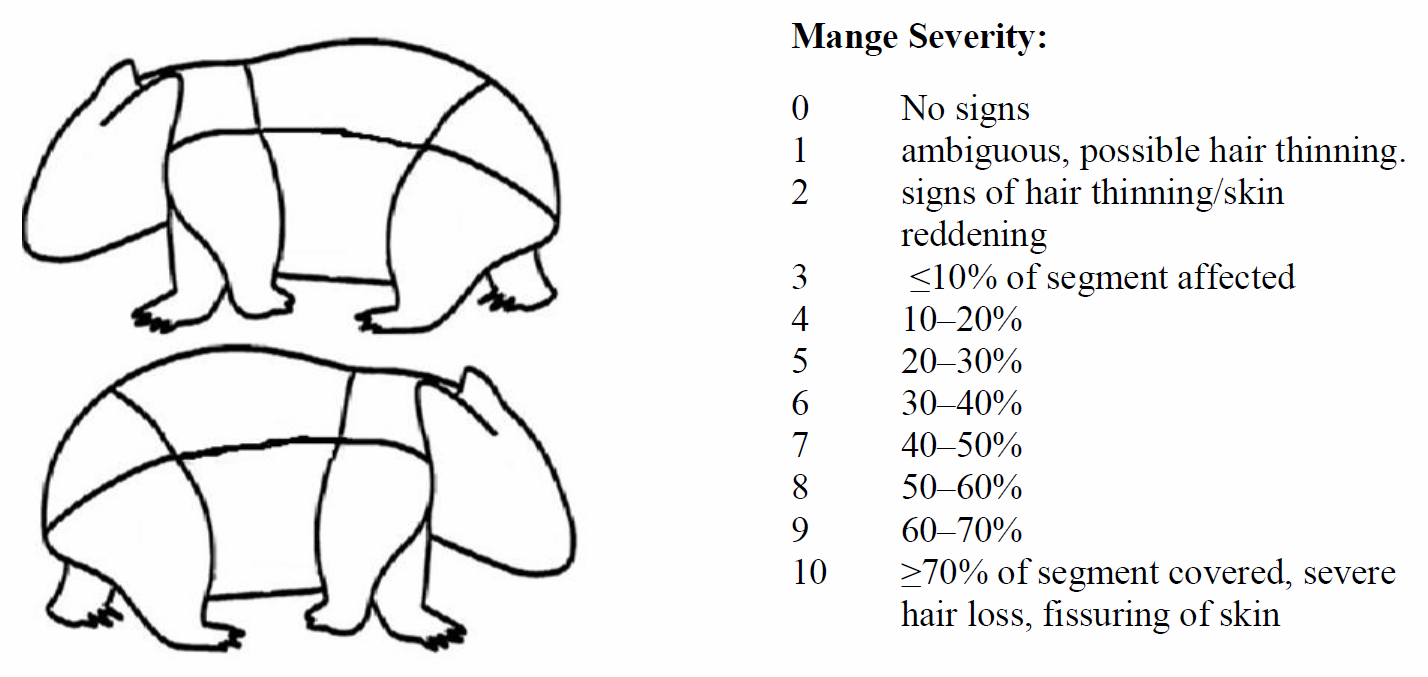

Supplement: S1 Text — Each segment was allocated a number (0–10) and the average of all segments gave the overall mange serverity score for each individual. Based on diagram in [33]. (TIF) [file pone.0149749.s002.tif]
